# A systematic review protocol of timing, efficacy and cost effectiveness of upper limb therapy for motor recovery post-stroke

**DOI:** 10.1186/s13643-019-1093-6

**Published:** 2019-07-25

**Authors:** Kathryn S. Hayward, Sharon F. Kramer, Vincent Thijs, Julie Ratcliffe, Nick S. Ward, Leonid Churilov, Laura Jolliffe, Dale Corbett, Geoffrey Cloud, Tina Kaffenberger, Amy Brodtmann, Julie Bernhardt, Natasha A. Lannin

**Affiliations:** 1 Melbourne School of Health Sciences, University of Melbourne, Parkville, Melbourne, Australia; 2 AVERT Early Rehabilitation Research Group, Stroke Theme, Florey Institute of Neuroscience and Mental Health, Parkville, Melbourne, Australia; 3 NHMRC CRE in Stroke Rehabilitation and Brain Recovery, University of Melbourne, Heidelberg, Melbourne, VIC 3084 Australia; 4 Stroke Theme, Florey Institute of Neuroscience and Mental Health and Neurology Department, Austin Health, Heidelberg, Melbourne, VIC Australia; 5 College of Nursing and Health Sciences, Flinders University, Adelaide, Australia; 6 UCL Institute of Neurology and The National Hospital for Neurology and Neurosurgery, London, UK; 7 Melbourne Medical School, University of Melbourne, Parkville, Melbourne, Australia; 8 College of Science, Health and Engineering, La Trobe University, Bundoora, Melbourne, VIC Australia; 9 Occupational Therapy Department, Alfred Health, Prahran, Melbourne, VIC Australia; 10 Department of Cellular and Molecular Medicine, University of Ottawa and Canadian Partnership for Stroke Recovery, Ottawa, Canada; 11 Department of Neurology, Alfred Health, Prahran, Melbourne, VIC Australia; 12 The Department of Clinical Neuroscience, Central Clinical School, Monash University, Melbourne, Australia; 13 Melbourne Dementia Research Centre, Florey Institute of Neuroscience and Mental Health, Heidelberg, Melbourne, VIC Australia; 14 Neurology Department, Austin Health, Heidelberg, Melbourne, VIC Australia; 15 Eastern Clinical Research Unit and Neurology Department, Eastern Health, Melbourne, VIC Australia; 16 School of Allied Health (Occupational Therapy), La Trobe University, Bundoora, Melbourne, VIC Australia

**Keywords:** Stroke, Upper limb, Systematic review, Protocol, Therapy, Rehabilitation, Recovery

## Abstract

**Background:**

Improving upper limb (UL) motor recovery after stroke represents a major clinical and scientific goal. We aim to complete three systematic reviews to estimate the (1) association between time to start of UL therapy and motor recovery, (2) relative efficacy of different UL therapy approaches post-stroke and (3) cost-effectiveness of UL therapy interventions.

**Methods:**

We have designed a systematic review protocol to address three systematic review questions that were each registered with PROSPERO. The search will be conducted in MEDLINE, EMBASE, and Cochrane Controlled Register of Trials. We will include randomised controlled trials, non-randomised clinical trials, before-after studies and observational studies of adult stroke survivors with an average stroke onset < 6 months, undergoing hospital-based therapy to improve UL function. Eligible interventions will aim to promote UL functional recovery. Two reviewers will independently screen, select and extract data. Study risk of bias will be appraised using appropriate tools. Clinical measures of motor recovery will be investigated (primary measure Fugl Meyer UL assessment), as well as measures of health-related quality of life (primary measure EQ-5D) and all cost-effectiveness analyses completed. Secondary outcomes include therapy dose (minutes, weeks, repetitions as available) and safety (i.e. adverse events, serious adverse events). A narrative synthesis will describe quality and content of the evidence. If feasible, we will conduct random effects meta-analyses where appropriate.

**Discussion:**

We anticipate the findings of this review will increase our understanding of UL therapy and inform the generation of novel, data-driven hypotheses for future UL therapy research post-stroke.

**Systematic review registration:**

PROSPERO, http://www.crd.york.ac.uk/PROSPERO/display_record.php?ID=CRD42018019367, http://www.crd.york.ac.uk/PROSPERO/display_record.php?ID=CRD42018111629, http://www.crd.york.ac.uk/PROSPERO/display_record.php?ID=CRD42018111628.

**Electronic supplementary material:**

The online version of this article (10.1186/s13643-019-1093-6) contains supplementary material, which is available to authorized users.

## Background

Up to 80% of stroke survivors have upper limb impairment early after stroke [1–3], and few demonstrate complete functional recovery at 6 months post-stroke [1, 4]. Upper limb rehabilitation trials designed to improve recovery rates have been largely unsuccessful. As a result, the burden of upper limb impairment after stroke remains high [5]. Therefore, understanding how to improve the potential for recovery of upper limb function remains a major scientific, clinical and patient priority [6, 7].

Why has it proved difficult to improve upper limb recovery after stroke? Upper limb therapy provided clinically is described as too little, too late in the recovery timeline [8]. Recent high-profile clinical trials of upper limb rehabilitation have evaluated relatively low amounts of therapy (13 to 36 h total or ~ 30 min/day), e.g. [9, 10] and/or enrolled patients relatively late post-stroke (i.e. > 6 months post-stroke), e.g. [11]. It remains unclear whether these trials were equivocal because the therapy was ineffective in terms of *how much*, *when* or *what* was delivered. Several syntheses have highlighted the importance of how much therapy; defining 30 min/day as too low [12], and 2 h per day as an emerging therapy threshold [13]. To date, no systematic review has examined the impact of the timing after stroke when rehabilitation is delivered.

Current clinical practice guidelines recommend that stroke patients start therapy as soon as possible post-stroke [14]. The rationale for early upper limb therapy post-stroke is supported by robust preclinical (animal) evidence, where it is well established that there is a sensitive window of heightened plasticity starting around 5 days after ischaemic stroke [15, 16]. In rodents, upper limb reaching therapy that is commenced at the beginning of the sensitive window capitalises on the injury-induced plasticity environment to produce gains in reaching that are quantitatively and qualitatively superior to those achieved when therapy of the same dose and duration is started later (around 14 and 30 days post-stroke) [15]. This underpins the rationale that timing of therapy is critically important. In humans, the post-stroke sensitive window is proposed to begin within the first 10 days post-stroke [17] and to extend out to 3 and even 6 months post-stroke [1, 4, 18]. The wide sensitive window of 6 months post-stroke needs to be better understood to guide recommendations around the critical time window to deliver upper limb therapy. A handful of upper limb trials have commenced training ≤ 10 days post-stroke [10, 19]. None have directly compared the effects of starting training early compared to a later time point. *This is a major gap*, which can be probed by looking at the functional gains demonstrated in previous upper limb studies that have started therapy and documented outcomes within the potential sensitive window, the first 6 months post-stroke.

The majority of completed upper limb therapy trials have tested what therapy approach to provide. Despite this, the ideal upper limb therapy intervention(s) that can improve recovery after stroke remains largely unknown. Previous Cochrane systematic reviews of specific therapy approaches have demonstrated an evidence gap, which highlights limited and/or insufficient evidence for upper limb therapy approaches that span repetitive task training [20], constraint-induced movement therapy [21], electromechanical and robot-assisted therapy [22] and hands-on-therapy interventions [23]. No systematic review has compared the relative benefit of different treatment approaches tested within clinical trials. While a previous review provided a summary of individual therapy approaches that started early post-stroke [24], the authors did not compare outcomes across therapy approaches. Direct comparison between different therapy approaches is also seldom performed in a single study due to sample size and cost constraints. Completing a review that directly compares different intervention approaches may help to establish the relative efficacy of each approach, as well as a potential hierarchy of treatment approaches to target in future trials.

The amount of upper limb therapy provided after stroke is currently pragmatic (i.e. based on available resources and what can be comfortably delivered within existing health services) rather than what might work (i.e. aspirational to match best scientific evidence). Delivery of more therapy is considered to be cost-ineffective (e.g. staffing, environment). However, consideration of the health economic arguments for optimum upper limb therapy is rarely mounted but are warranted to inform best clinical practice and policy decisions.

To address these gaps, we aim to identify relevant upper limb therapy studies that aim to improve functional recovery post-stroke and include participants of an average stroke onset < 6 months who are undergoing hospital-based therapy. We have designed a systematic review protocol to address three research questions that have been prospectively registered with PROSPERO. Systematic reviews are resource- and time-intensive, using data from a single search strategy to inform multiple questions is one way to mitigate such issues. We plan to address the following study objectives:

1. Estimate the association between time to start upper limb therapy and outcome, taking into consideration dose of therapy;
2. Estimate the relative efficacy of different upper limb therapy approaches post-stroke, taking into consideration time to start and dose of therapy; and
3. Estimate the cost-effectiveness of upper limb therapy interventions.

## Methods and design

The present protocol is reported in accordance with the Preferred Reporting Items for Systematic Reviews and Meta-Analyses Protocols (PRISMA-P) statement [25–27] (see PRISMA-P checklist in Additional file 1). This protocol covers three research questions that will be answered from one systematic search strategy. All research questions have been prospectively registered on the International Prospective Register of Systematic Reviews (PROSPERO): timing (registration number, CRD42018019367), therapy efficacy (registration number, CRD42018111629) and cost-effectiveness (registration number, CRD42018111628).

### Search strategy for identification of relevant studies

Electronic searches will be conducted in MEDLINE (via Ovid), EMBASE (via Ovid) and Cochrane Controlled Register of Trials (CENTRAL). Hand searches of reference lists of included studies or identified relevant reviews will be employed. The search strategy will include terms related to stroke, upper limb function and movement, and therapy (see Additional file 1 for MEDLINE/EMBASE, which was adapted for CENTRAL). The only search strategy limit is ‘human’. Language limits of English, Dutch, French, German will be applied when screening studies.

### Eligibility

Studies will be selected according to the criterion outlined below.

#### Types of studies

We will select randomised controlled trials (RCTs), including cluster RCTs, controlled (non-randomised) clinical trials or cluster trials, before-after studies, and observational studies (prospective and retrospective cohort studies, case-control or nested case-control studies) which have a minimum of two waves of assessment, i.e. pre/baseline and post-intervention/follow up. We will exclude cross-sectional studies, case series, case reports, qualitative studies, surveys, protocols papers, conference proceedings and reviews. Data from any study design will be included in the timing systematic review; however, only RCTs will be included in the therapy efficacy. The cost-effectiveness systematic review will include studies reporting full economic evaluations using cost-effectiveness and/or cost-utility analysis methods incorporating both studies conducted alongside RCTs and health economic model-based analyses (e.g. deterministic decision analysis, Markov models, populated with data from the literature).

#### Participants

Studies that include adults (≥18 years) with a diagnosis of stroke (ischaemic or haemorrhagic, or at minimum 50% of the sample), average stroke onset < 6 months and undergoing hospital-based in- or out-patient therapy will be included.

#### Interventions

All studies of upper limb therapy (experimental or usual care intervention) with the aim to improve function will be included. Examples include bilateral arm training, biofeedback, Bobath approach, constraint-induced movement therapy, electrical stimulation, hands-on therapy (manual therapy techniques), repetitive task training, robotics, strength training, task-specific training, virtual reality, standard therapy [28]. As this review centres around upper limb *motor* therapy only, we will exclude

- Use pharmacological (e.g. recovery-promoting drugs), complimentary (e.g. acupuncture), non-invasive brain stimulation or priming intervention(s) (e.g. transcranial magnetic stimulation) in combination with or without therapy
- Have a primary focus to reduce secondary impairments, e.g. pain, contracture, spasticity, subluxation
- Focus on general motor recovery, e.g. activities of daily living
- Focus on non-motor impairments, e.g. sensory, hemispatial neglect with or without motor practice
- Do not include any upper limb therapy, e.g. mental/motor imagery alone

#### Comparisons or control

No restrictions will be made on the comparison or control group (e.g. attention control groups and active comparisons will be included).

#### Outcome measures

All clinical measures used to document upper limb recovery (change) [17] across two assessment time points, e.g. pre to post-intervention will be explored, as well as follow up if available to provide information about the sustainability of recovery gains. For the review of timing and efficacy, the clinical outcome that is considered to best reflect recovery of upper limb impairment is the Fugl Meyer Upper Limb (FMUL) [29]. This measure has been recommended by the international Stroke Rehabilitation and Recovery Roundtable as a core data element [29]. As this recommendation was made in 2017, many studies may report other measures. This includes measures of upper limb activity, such as Action Research Arm Test (ARAT), Box and Block Test (BBT), Wolf Motor Function Test (WMFT), Nine-Hole Peg Test (9HPT) and Motor Assessment Scale (MAS) [13]. Where multiple measures are published, the Fugl Meyer will be prioritised over all other measures (primary outcome), followed by timed measures (e.g. Box and Block Test) over observational measures (e.g. MAS). Timed measures will be prioritised over observational (or quality of movement) measures as they reduce examiner bias. Secondary outcomes of therapy dose (i.e. minutes, weeks, repetitions as available) and safety (i.e. reported adverse events and serious adverse events) will be considered (see data extraction for more details).

We will explore change in health-related quality of life within the cost-effectiveness review. We will extract any generic- or condition-specific preference or non-preference-based measures of health-related quality of life. Where multiple measures are published, we will prioritise incremental change in health-related quality of life measured using EuroQol (EQ-5D) [30, 31]. EQ-5D is our top priority as it is the recommended core data element from the International Stroke Rehabilitation and Recovery Roundtable and is most widely used in clinical research [29].

### Screening of studies

All studies identified by the search strategy will be uploaded to Covidence (https://www.covidence.org/ [32]) and duplicates removed. Two authors (KH/SK) will screen studies for eligibility based on title and abstract using predetermined criteria (see Table 1 for summary). Full text for all remaining studies will be retrieved and reviewed independently (KH/SK). Reports from the same study population will be linked to ensure that data from a particular population is only included once in analysis. Authors of studies may be contacted to collect data that is missing or to clarify details of the study to ensure appropriate study inclusion (e.g. regarding post-stroke time to recruitment). If a disagreement regarding eligibility occurs, it will be resolved by discussion and review of criteria amongst KH/SK. If not resolved, a third reviewer (one of NL or NW) will be involved to achieve a consensus. If still not resolved, a further two reviewers (two of JB/VT/DC) will be involved. If no consensus is reached, all reviewers will discuss the article, and if no consensus is achieved, it will be documented in the review. The results of the screening process will be provided in detail using a PRISMA study flow chart.

**Table 1:** Eligibility criteria

Inclusion	Exclusion
Adults (>17 years) with a diagnosis of stroke (ischaemic or haemorrhagic) and average stroke onset <6 months (or at least 50% of the sample has diagnosis of stroke within the time frame) undergoing hospital based (in or outpatient) rehabilitation	Pharmacological, complimentary, non-invasive brain stimulation or priming intervention(s) [delivered in conjunction with motor practice interventions(s) or alone, ie as a control].
Upper limb therapy (experimental or usual care intervention) with the aim to improve function	Interventions with the primary focus to reduce secondary impairments e.g., pain, contracture, spasticity, subluxation; that do not include any upper limb motor practice e.g., mental/motor imagery practice alone; or for general motor practice e.g., activities of daily living, or non-motor impairments e.g., sensory, hemispatial neglect.
A minimum of two waves of motor impairment or activity assessment i.e., pre and post intervention	
Study design of RCT, non RCT, cohort and observational, pre-post single group. Any control intervention eligible.	Single case, case series, qualitative, surveys, protocols, conference proceedings, cross-sectional, review, single session intervention
Languages: English, Dutch, French, German (SK/VT/TK fluent).	

### Data extraction

Two independent reviewers (of KH/TK/LJ/NL) will extract data using a predetermined data collection form. If queries or discrepancies regarding data extraction occur, these will be resolved by discussion between reviewers. If not resolved, an additional reviewer will extract the data (of VT/GC/AB). If no consensus is achieved, our statistician (LC) will review the paper and make a final verdict. The data extraction form will record information regarding the following:

1. Study details, i.e. authors, date, location of study and setting, design and stratification
2. Participants’ information, i.e. number of participants, age, sex, characteristics of stroke including type, severity of stroke and upper limb impairment
3. Clinical outcome measures, i.e. impairment and activity (per *outcome measures*)
4. Biomarker measures of the corticospinal tract, i.e. motor evoked potential status
5. Time post-stroke to recruitment, assessment and/or intervention start, i.e. days post-stroke
6. Dose of therapy, i.e. minutes, weeks and repetitions as available. We will extract minutes or repetitions per session, per day and total minutes and repetitions. Where both active dose and total dose (active and rest) are provided, both will be extracted and synthesised separately.
7. Schedule of therapy, i.e. frequency of sessions, weeks of therapy
8. Content of therapy, i.e. therapy approach, structure of therapy sessions
9. Safety, i.e. adverse events and serious adverse events as the total number
10. Economic evaluation, i.e. resource use associated with the intervention and estimating resources and costs, study perspective (e.g. health system, societal), time horizon, discount rate, currency, price data, assumptions, personal and societal, and health-related quality of life (per above *outcome measures* above), as well as the full economic evaluations using cost-effectiveness and/or cost-utility analysis and health economic model-based analyses
11. Results for clinical measures, i.e. means, standard deviations, coefficients, *p* values, effect size, medians, interquartile ranges
12. Missing/incomplete data to be followed up

The corresponding author of any study with missing or incomplete data will be contacted for further information.

### Risk of bias

We will use the Cochrane Risk of Bias tool to examine bias across five domains for randomised controlled trials [33]. For other designs, we will use the ROBINS-I tool [34]. Two reviewers (of JB/NW/VT) will use the appropriate tool to independently rate each included study. If queries or discrepancies regarding data extraction occur, these will be resolved by discussion between reviewers. If not resolved, a further two authors (GC/AB) will complete the risk of bias tool. If no consensus is achieved, our statistician (LC) will review the paper and make a final verdict.

### Strategy for data synthesis

Our systematic review results will be reported by describing study characteristics, participant characteristics and outcome results. For all outcomes considered, we will present summary data for each therapy group and effect estimates and confidence intervals as feasible. We will also describe our literature search results, as well as the methodological quality and risk of bias results using tables, figures and text. Strength of the evidence will be determined using GRADE as appropriate [35]. We will evaluate whether we have sufficient data to conduct random effects meta-analysis. We will also ensure that the body of literature is sufficiently homogenous in terms of clinical (e.g. patient characteristics), methodological (e.g. study design) and statistical (e.g. forest plot consistency) characteristics. For example, we will use our clinical insight to assess for clinical heterogeneity, methodologists will assess for methodological heterogeneity, and statistical heterogeneity will be calculated. We will try to explain potential heterogeneity via subgroup analysis and meta-regression analysis, as described below.

#### Timing

We will explore the distribution of the data and allocate cohorts to timing categories based on mean/median group values. Our framework for time window classification is the recent Stroke Rehabilitation and Recovery Roundtable recommendations, as the chosen time-windows are linked to our current understanding of the neurobiology of recovery [17]:

- Hyperacute, ≤ 24 h post-stroke
- Acute, > 24 h but ≤ 7 days post-stroke
- Early subacute, > 7 days but ≤ 3 months post-stroke
- Late subacute, > 3 months but ≤ 6 months post-stroke

If data are appropriate for quantitative synthesis, we will pool effects across studies within each time window (subgroups) using random effects meta-analysis models to report standardised mean difference (95% CI). This will accommodate both our primary outcome of interest (Fugl Meyer), as well as other outcome measures that may be used. Standard deviation to inform the analysis will be extracted from text, calculated from available data in text or by contacting the primary author. We will also calculate measures of heterogeneity and consistency as appropriate. If data are appropriate, the association of timing with estimated effects on functional outcomes using a random effects meta-regression analyses and a sensitivity analysis of studies assessed as low risk of bias will be completed.

#### Clinical efficacy

We will allocate studies to therapy approaches consistent with a recent Cochrane review [28], with inappropriate categories removed consistent with our eligibility criteria, e.g. recovery-promoting drugs and non-invasive brain stimulation. Intervention approach categories of interest are bilateral arm training, biofeedback, Bobath approach, constraint-induced movement therapy, electrical stimulation, hands-on therapy (manual therapy techniques), repetitive task training, robotics, strength training, task-specific training, virtual reality, standard therapy category (i.e. control group) and others. If data are appropriate, we will attempt conducting a network meta-analysis (NMA). This analysis is particularly useful when there is a lack of head-to-head studies or when both relevant head-to-head and standard treatment-controlled studies exist. The network meta-analysis approach allows ranking of interventions. Key considerations that will be evaluated on completion of data extraction for NMA will be handling of multi-arm trials, variance structures and assessment of fit and consistency. This analysis will be conducted in Stata and/or R, and median rankings (or point estimates) will be calculated using a random effects model that makes use of all available direct and indirect data. The degree of uncertainty for all point estimates will be reported as 95% confidence/credible intervals (CIs).

#### Cost-effectiveness

We will use the CHEERS statement to examine the reporting quality of the identified economic evaluations [36]. If possible, we will pool effects across studies using random effects meta-analysis models as appropriate for specific outcomes and the data available for analysis. Key considerations for pooled analysis will be the number of studies identified, their quality and consistency of cost and outcomes.

### Ethics, amendments and dissemination

We will use only secondary de-identified data to address the three research questions; therefore, ethics approval is not required. Any protocol amendments will be tracked against our PROSPERO record and outlined in the final publication. The findings of the three reviews will be disseminated through presentation at appropriate forums and conferences. The completed reviews will be submitted for publication in peer-reviewed journals. Findings will be translated to inform the development of upper limb therapy protocols that will be tested in future clinical trials.

## Discussion

The described protocol represents a novel approach to undertaking a systematic review. By using a broad search strategy, we can address several critical research questions simultaneously. An appropriately designed search strategy is likely to yield large volumes of data and will require considerable time to determine eligibility and extract relevant data. The proposed approach to completing a systematic review will minimise research waste, which is critical given the time taken to complete systematic reviews. Furthermore, the outputs will synthesise past research to generate novel, data-driven hypotheses for future research that aims to define a new therapy pathway(s) to promote upper limb recovery. While each review may yield the answers to our questions, there is the likelihood that they will demonstrate that we do not yet have the appropriate data to allow clinicians to make evidence-based decisions about hospital-based upper limb therapy within the first 6 months post-stroke. This finding is as important as yielding all the answers and will form the foundation for designing studies that can answer these important research questions in stroke rehabilitation and recovery.

The methods and results of our systematic reviews will be reported following the PRISMA statement and their relevant extensions [25–27]. While this review will be completed with the upmost of care, there are some limitations. It is well documented that a large range of upper limb outcome measures are reported in research [37] which prevents individual patient data analysis. Further, there may be missing data that will impact data collation, comparison across studies and pooled analyses. We acknowledge language restrictions beyond English, German, French and Dutch. We do not intend to hand-search conference proceedings. In addition, the observational nature of the subgroup analyses means they should be interpreted with caution, as it is known that subgroup analyses can be less highly powered than analyses for main effects.

## Additional file

Additional file 1: Search strategy. (DOCX 14 kb)

## Data Availability

Not applicable.

## Abbreviations

9HPT: Nine-Hole Peg Test
ARAT: Action Research Arm Test
BBT: Box and Block Test
EQ-5D: EuroQoL 5-dimension
FMUL: Fugl Meyer Upper Limb
HR-QoL: Health-related quality of life
MAS: Motor Assessment Scale
RCT: Randomised controlled trial
WMFT: Wolf Motor Function Test
