# Supplementary material for: A systematic review protocol of timing, efficacy and cost effectiveness of upper limb therapy for motor recovery post-stroke
Source: Syst Rev. 2019 Jul 25;8:187. doi: 10.1186/s13643-019-1093-6 (PMC6657039; doi:10.1186/s13643-019-1093-6)
Supplement: Supplementary file 1 — Search strategy. (DOCX 14 kb) [file 13643_2019_1093_MOESM1_ESM.docx]

**Search strategy**

MEDLINE & EMBASE, which was adapted for CENTRAL

1. exp Cerebrovasular Disorders/
2. stroke$.tw.
3. cva$.tw.
4. cerebrovasuclar$.tw.
5. cerebral vascular$.tw.
6. (poststroke or post-stroke or cerebrovasc$ or brain vasc$ or cerebral vasc$ or apoplex$ or SAH).tw.
7. ((brain$ or cerebr$ or cerebell$ or intracran$ or intracerebral or vertebrovasilar) adj5 (isch?emi$ or infarct$ or thrombo$ or emboli$ or occlus$)).tw.
8. ((brain$ or cerebr$ or cerebell$ or intracerebral or intracranial or subarachnoid) adj5 (haemorrhage$ or hemorrhage$ or haematoma$ or hematoma$ or bleed$)).tw.
9. hemiplegia/ or exp paresis/
10. (hemipleg$ or hemipar$ or paresis or paretic).tw.
11. 1 or 2 or 3 or 4 or 5 or 6 or 7 or 8 or 9 or 10
12. exp Physical Therapy Modalities/
13. physical therapy.mp.
14. Physiotherapy.mp.
15. Occupational Therapy/
16. rehabilitat$.mp.
17. Rehabilitation/
18. motor relearn$.mp.
19. bobath.mp.
20. 12 or 13 or 14 or 15 or 16 or 17 or 18 or 19
21. exp upper extremity/
22. (upper limb$ or upper extremit$ or arm or shoulder or hand or axilla or elbow$ or forearm$ or finger$ or wrist$).tw.
23. 21 or 22
24. 11 and 20 and 23
25. Control for Humans
